# Using a risk model for probability of cancer in pulmonary nodules

**DOI:** 10.1111/1759-7714.13991

**Published:** 2021-05-11

**Authors:** Si‐Qi Liu, Xiao‐Bin Ma, Wan‐Mei Song, Yi‐Fan Li, Ning Li, Li‐Na Wang, Jin‐Yue Liu, Ning‐Ning Tao, Shi‐Jin Li, Ting‐Ting Xu, Qian‐Yun Zhang, Qi‐Qi An, Bin Liang, Huai‐Chen Li

**Affiliations:** ^1^ Department of Respiratory and Critical Care Medicine, Shandong Provincial Hospital, Cheeloo College of Medicine Shandong University Jinan China; ^2^ Cheeloo College of Medicine Shandong University Jinan China; ^3^ Shandong Medical Imaging Research Institute, Cheeloo College of Medicine, Shandong University Jinan China; ^4^ Department of Medical Imaging, Shandong Provincial Hospital, Cheeloo College of Medicine Shandong University Jinan China; ^5^ Department of Intensive Care Unit Shandong Provincial Third Hospital Jinan China; ^6^ Department of Respiratory and Critical Care Medicine Beijing Hospital Beijing China; ^7^ Graduate School of Peking Union Medical College Chinese Academy of Medical Sciences and Peking Union Medical College Beijing China; ^8^ College of Traditional Chinese Medicine Shandong University of Traditional Chinese Medicine Jinan China

**Keywords:** decision tree, logistics regression, lung cancer, pulmonary nodules

## Abstract

**Background:**

Considering the high morbidity and mortality of lung cancer and the high incidence of pulmonary nodules, clearly distinguishing benign from malignant lung nodules at an early stage is of great significance. However, determining the kind of lung nodule which is more prone to lung cancer remains a problem worldwide.

**Methods:**

A total of 480 patients with pulmonary nodule data were collected from Shandong, China. We assessed the clinical characteristics and computed tomography (CT) imaging features among pulmonary nodules in patients who had undergone video‐assisted thoracoscopic surgery (VATS) lobectomy from 2013 to 2018. Preliminary selection of features was based on a statistical analysis using SPSS. We used WEKA to assess the machine learning models using its multiple algorithms and selected the best decision tree model using its optimization algorithm.

**Results:**

The combination of decision tree and logistics regression optimized the decision tree without affecting its AUC. The decision tree structure showed that lobulation was the most important feature, followed by spiculation, vessel convergence sign, nodule type, satellite nodule, nodule size and age of patient.

**Conclusions:**

Our study shows that decision tree analyses can be applied to screen individuals for early lung cancer with CT. Our decision tree provides a new way to help clinicians establish a logical diagnosis by a stepwise progression method, but still needs to be validated for prospective trials in a larger patient population.

## Graphical abstract

Considering the high morbidity and mortality of lung cancer and the high incidence of pulmonary nodules, it is of great significance to clearly distinguish between benign and malignant lung nodules at early stages. However, what kind of lung nodules are more prone to lung cancer is still a worldwide problem. In this study, 458 cases with pulmonary nodules data were collected from Shandong, China. We assessed the clinical characteristics and low‐dose computed tomography (LDCT) imaging features among pulmonary nodules in patients who had undergone video‐assisted thoracoscopic surgery (VATS) lobectomy from 2013 to 2018 to determine the machine learning models using multiple algorithms and select the best decision tree model. Our study shows that decision tree analyses can be applied to screen individuals for early lung cancer with CT. Our decision tree provides a new way to help clinicians establish a logical diagnosis by a stepwise progression method, but still needs to be validated for prospective trials in a larger patient population.

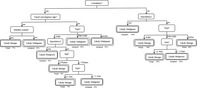

## INTRODUCTION

Lung cancer shows the highest morbidity and mortality of all cancers in both sexes combined worldwide with a large proportion of patients being diagnosed at an advanced stage of disease.^1^

Previous studies^2^, ^3^, ^4^ have demonstrated that computed tomography (CT) is recommended by US guidelines for high‐risk individuals to reduce lung cancer mortality because more early‐stage lung cancers can be diagnosed with the assistance of CT and more invasive procedures can be implemented. Owing to the widespread availability of CT screening, more and more lung nodules are being diagnosed in a timely manner, so that the risk of lung cancer screening programs is surgical resection performed for intent to cure malignant disease in patients without lung cancer.^5^ As medical technology advances, patients undergoing video‐assisted thoracoscopic surgery (VATS) lobectomy have been reported to exhibit lower probability of readmission, pneumonia, and postoperative blood transfusion compared with those undergoing open lobectomy;^6^ however, it is not only a waste of medical resources in patients with benign nodules undergoing surgery, but incalculable harm can be caused to patients' body and mind. Therefore, it is crucial to improve the diagnostic accuracy of lung cancer and reduce unnecessary surgery. Although many studies^7^, ^8^, ^9^, ^10^ have previously used different methods to analyze differences in the CT imaging characteristics between benign nodules and lung cancer patients, how to analyze benign and malignant nodules remains controversial.

Big data mining technology has opened up a new era in which guidelines and characteristics of many things are readily available from a mass of basic data.^11^ In lung cancer, mixed models combining multiple factors have been shown to provide excellent prognostic benefits.^12^, ^13^ At present, many studies have tried to establish models to achieve the intelligent identification of benign and malignant nodules, and have shown that machine learning plays an irreplaceable role in disease diagnosis.^14^, ^15^

In this study, we performed a retrospective analysis of patients with lung nodules undergoing VATS lobectomy which aimed to (i) compare the clinical features and image characteristics of pulmonary benign and malignant nodules, (ii) compare several common machine learning models from multiple aspects and (iii) provide a new method for clinicians to distinguish benign from malignant pulmonary nodules.

## METHODS

### Patient selection

**FIGURE 1:**
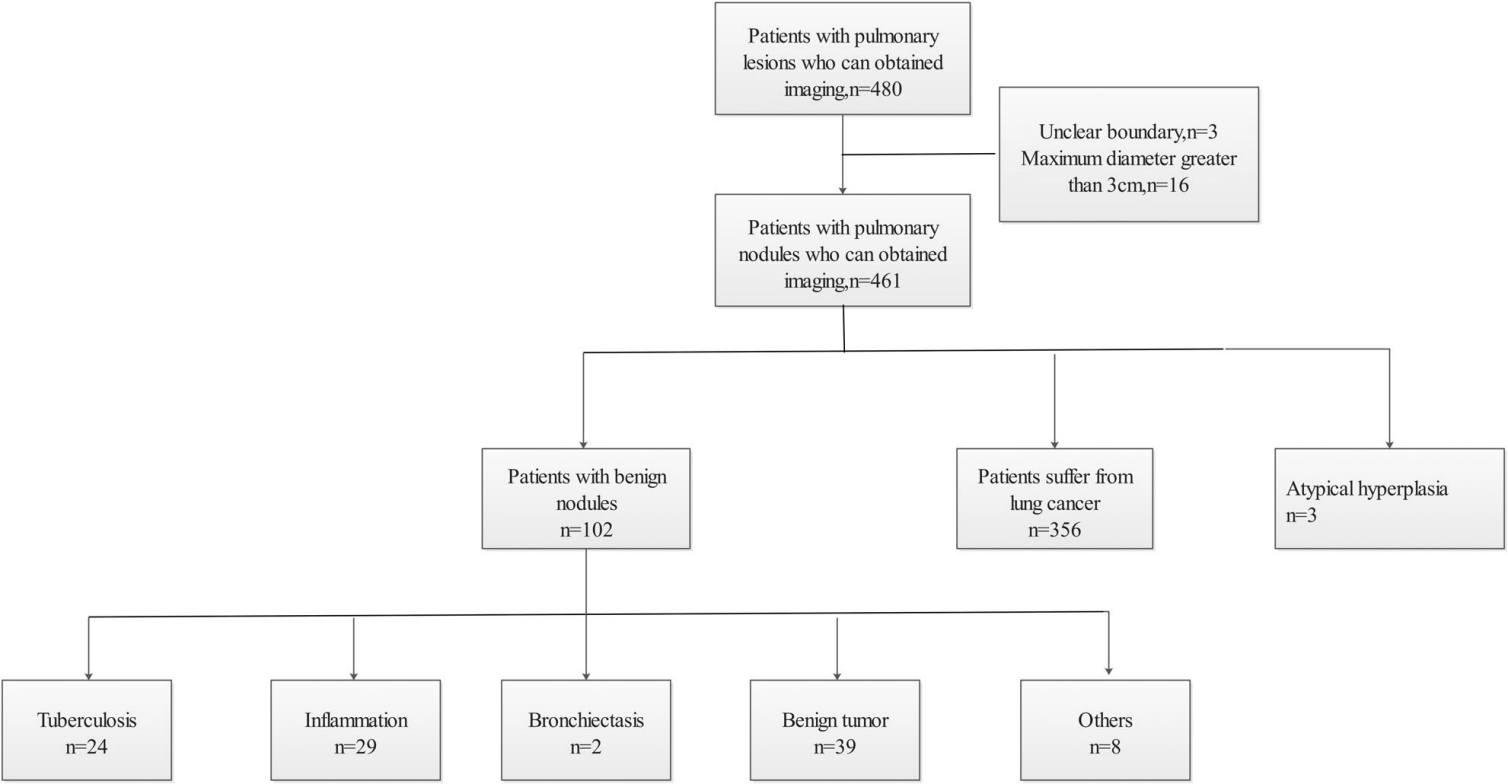
Pathological results of lung nodules patients that were underwent VATS lobectomy from January 2013 to November 2018 in Shandong Provincial Hospital

A retrospective analysis was conducted of 480 (Figure 1) patients with lung nodules who had undergone VATS lobectomy from January 2013 to November 2018 in Shandong Provincial Hospital, China. Weobtained definitive pathological results following surgery, which allowed us to proceed to further studies. First, we preliminarily excluded nodules greater than 3 cm in the longest length (16 cases) from the imaging reports. We also excluded nodules with unclear boundaries that could not be studied further (three cases). In addition, nodules confirmed by pathology as atypical hyperplasia (three cases) was also excluded. Thus, there were 458 cases (102 cases of benign nodules and 356 cases of malignant nodules) in the study. Before the patients underwent lobectomy via video‐assisted thoracoscopic surgery (VATS), we performed auxiliary examinations such as craniocerebral magnetic resonance imaging (MRI), abdominal ultrasound, and also positron emission tomography/computed tomography (PET/CT). If tumors outside the lung were found, surgery was not performed; therefore the samples in this study did not include those patients with metastatic lung cancer.

### Patient characteristics

The clinical characteristics of all patients were derived from the electronic medical record system of the hospital, including gender, age, profession, smoking history, drinking history and family history of cancer. We evaluated all chest CTs for each patient within our picture archiving and communication system (PACS), and both radiologists who had received special training in chest radiology described the characteristics of the nodules without knowing the pathological results of the nodules. Conclusions were made with consensus. Our CT cases were obtained using Somatom Definition Flash CT, Somatom Definition Edge CT and Somatom Force CT Scanner (Siemens). At our institution, chest CT reconstruction protocols include 1‐, 1.25‐ and 5‐mm axial slices. A previous study^16^ has stated that various viewing techniques have similar detection rates when experienced observers focus on nodule detection. Sagittal and coronal reconstructions are routinely obtained.

Documented characteristics of the nodules included their maximum diameter, location in the lung, signs of lobulation, spiculation, satellite nodule, vessel convergence sign, pleural indentation, if there was a distinct boundary, and types determined by density (solid, ground‐glass nodules or part‐solid). A solitary pulmonary nodule (SPN) is defined as a round opacity that is at least moderately well circumscribed and no larger than 3 cm in diameter.^17^ SPNs include solid and subsolid nodules, and subsolid nodules include ground‐glass nodules (GGNs) and part‐solid nodules.^18^ Ground‐glass nodules (GGNs) are nuanced nodular opacities that do not obscure underlying bronchovascular structures of the lung.^19^

### Statistical analysis

Categorical clinical characteristics include gender (male or female), age (0–45, 45–60, 60+), profession (others, workmen, labourers, office clerk), smoking (no/yes), drinking (no/yes), and family history of cancer (no/yes). They were compared between benign and malignant nodules using logistic regression analyses. In addition, categorical variables such as nodule size (<0.6 cm, 0.6–1.0 cm, 1.0–2.0 cm, 2.0–3.0 cm), nodule location, lobular (no/yes), spiculation (no/yes), boundary (no/yes), satellite nodule (no/yes), vessel convergence sign (no/yes), pleural indentation (no/yes) and nodule type (solid, GGNs or part‐solid) were added. Univariable analysis and a multivariable logistic model were applied to explore the risk factors of lung cancer among patients with pulmonary nodules diagnosed by CT. Variables which had statistical significance in the univariable analysis were included in the multivariate analysis, and the Holm‐Bonferroni correction was subsequently applied to factors with *p*‐values <0.05. All logistic models were performed using SPSS software (version 20.0). The diagnostic performance of the predictive model was calculated by the receiver operating characteristic (ROC) curve analysis. In addition, we compared multiple indicators of four common machine learning models (naivebayes, support vector machines, decision tree and random forests). All models were developed within WEKA (Waikato Environment for Knowledge Analysis) 3.8.3 (The University of Waikato, Hamilton, NZ). Moreover, the support vector machines (SVM) used a sequential minimal optimization (SMO) algorithm and the decision tree used a cost‐sensitive version of J48, an implementation of the C4.5 algorithm. The area under the curve (AUC) of models were analyzed using one‐way ANOVA and Tamhane's T2 post hoc at a significance level of α = 0.001.

## RESULTS

### Clinical characteristics

During the study period, a total of 458 patients with pulmonary nodules who had undergone VATS lobectomy were included; 102 (22.27%) were cases with benign nodules and 356 (77.73%) were lung cancer cases. There was a narrow gender gap between the two sets of data, and the proportion of female cases was 40.20% (41/102) and 51.12% (182/356), respectively (*p* > 0.05). In the univariate analysis, patients with pulmonary nodules who were male (OR: 0.64; 95% CI: 0.41–1.01), workmen (OR: 0.99; 95% CI: 0.41–2.37), or with a history of smoking (OR: 0.80; 95% CI: 0.51–1.27), alcohol use (OR:0.85; 95% CI, 0.51–1.44) or with a family history of cancer (OR: 0.71; 95% CI: 0.42–1.21) had a higher risk of benign nodules. In contrast, laborers (OR: 1.28; 95% CI: 0.67–2.42) or office clerks (OR: 1.76; 95% CI: 0.92–3.35) were risk factors for lung cancer. However, none of these were statistically significant (*p* > 0.05). The only factor that was statistically significant was age, and older people (45–60 years, OR: 2.06; 95% CI: 1.31–3.76; *p* = 0.018, or>60 years, OR: 5.00; 95% CI: 2.56–9.75; *p* < 0.001) had a higher risk of suffering from lung cancer. The mean age of patients with benign nodules (52.35 ± 10.86 years) was younger than lung cancer cases (58.91 ± 9.69 years). The age distribution of benign nodules cases and lung cancer cases was about 24.51% and 9.83% under age 45, 51.96% and 42.98% between the ages of 45 and 60 years, and 23.53% and 47.19% in patients aged more than 60 years, respectively (Table 1).

**TABLE 1:** Clinical and nodule characteristics of 458 patients with pulmonary nodules diagnosed with computer tomography (CT) who underwent video‐assisted thoracoscopy (VATS) lobectomy in Shandong Province Hospital, China

Variable	Benign nodule *n* = 102 (22.27%)	Lung cancer *n* = 356 (77.73%)	Total *n* = 458	Univariable analysis		Multivariable analysis	
				OR (95%CI)	*p*‐value	OR (95% CI)	*p*‐value (Holm‐Bonferroni)
Sex							
Female	41 (40.20%)	182 (51.12%)	223 (48.69%)	Reference	Reference		
Male	61 (59.80%)	174 (48.88%)	235 (51.31%)	0.64 (0.41–1.01)	0.053		
Age (years)							
Average	52.35 ± 10.86	58.91 ± 9.69	57.45 ± 10.33				
≤45	25 (24.51%)	35 (9.83%)	60 (13.10%)	Reference	Reference	Reference	Reference
45–60	53 (51.96%)	153 (42.98%)	206 (44.98%)	2.06 (1.31–3.76)	0.018^*^	1.80 (0.85–3.82)	0.125
>60	24 (23.53%)	168 (47.19%)	192 (41.92%)	5.00 (2.56–9.75)	<0.001^***^	3.30 (1.46–7.48)	0.004^**^
Profession							
Others	19 (18.63%)	49 (13.76%)	68 (14.85%)	Reference	Reference		
Workmen	11 (10.78%)	28 (7.87%)	39 (8.52%)	0.99 (0.41–2.37)	0.997		
Laborers	38 (37.25%)	125 (35.11%)	163 (35.59%)	1.28 (0.67–2.42)	0.458		
Office clerk	34 (33.33%)	154 (43.26%)	188 (41.05%)	1.76 (0.92–3.35)	0.088		
Smoking							
No	64 (62.75%)	241 (67.70%)	305 (66.59%)	Reference	Reference		
Yes	38 (37.25%)	115 (32.30%)	153 (33.41%)	0.80 (0.51–1.27)	0.350		
Drinking							
No	78 (76.47%)	282 (79.21%)	360 (78.60%)	Reference	Reference		
Yes	24 (23.53%)	74 (20.79%)	98 (21.40%)	0.85 (0.51–1.44)	0.552		
Family history of cancer							
No	78 (76.47%)	282 (79.21%)	370 (80.79%)	Reference	Reference		
Yes	24 (23.53%)	74 (20.79%)	88 (19.21%)	0.71 (0.42–1.21)	0.211		
Nodule size‐cm							
Average	1.59 ± 0.64	1.84 ± 0.63	1.78 ± 0.64				
<0.6	5/8 (62.50%)	3/8 (37.50%)	8/458 (1.75%)	Reference	Reference	Reference	Reference
0.6–1.0	19/60 (31.67%)	41/60 (68.33%)	60/458 (13.10%)	3.60 (0.78–16.63)	0.101	2.26 (0.38–13.35)	0.370
1.0–2.0	56/231 (24.24%)	175/231 (75.76%)	231/458 (50.44%)	5.21 (1.21–22.49)	0.027^*^	3.42 (0.62–19.00)	0.160
2.0–3.0	22/159 (13.84%)	137/159 (86.16%)	159/458 (34.72%)	10.38 (2.32–46.54)	0.002^**^	6.23 (1.07–36.29)	0.042^*^
Nodule location							
Right upper lobe	25/129 (19.38%)	104/129 (80.62%)	129/458 (28.17%)	Reference	Reference		
Right middle lobe	8/38 (21.05%)	30/38 (78.95%)	38/458 (8.30%)	0.90 (0.37–2.20)	0.820		
Right lower lobe	18/90 (20.00%)	72/90 (80.00%)	90/458 (19.65%)	0.96 (0.49–1.89)	0.910		
Left upper lobe	25/107 (23.36%)	82/107 (76.64%)	107/458 (23.36%)	0.79 (0.42–1.47)	0.456		
Left lower lobe	26/94 (27.66%)	68/94 (72.34%)	94/458 (20.52%)	0.63 (0.34–1.18)	0.148		
Lobulation							
No	27/45 (60.00%)	18/45 (40.00%)	45/458 (9.83%)	Reference	Reference	Reference	Reference
Yes	75/413 (18.16%)	338/413 (81.84%)	413/458 (90.17%)	6.76 (3.54–12.91)	<0.001^***^	3.34 (1.38–8.12)	0.008^**^
Spiculation							
No	79/246 (32.11%)	167/246 (67.89%)	246/458 (53.71%)	Reference	Reference	Reference	Reference
Yes	23/212 (10.85%)	189/212 (89.15%)	212/458 (46.29%)	3.89 (2.34–6.47)	<0.001^***^	3.15 (1.71–5.82)	<0.001^***^
Boundary							
Distinct	98/417 (23.50%)	319/417 (76.50%)	417/458 (91.05%)	Reference	Reference		
Indistinct	4/41 (9.76%)	37/41 (90.24%)	41/458 (8.95%)	2.84 (0.99–8.17)	0.053		
Satellite nodule							
No	97/452 (21.46%)	355/452 (78.54%)	452/458 (98.69%)	Reference	Reference	Reference	Reference
Yes	5/6 (83.33%)	1/6 (16.67%)	6/458 (1.31%)	0.06 (0.01–0.47)	0.008^**^	0.05 (0.01–0.44)	0.007^**^
Vessel convergence sign							
No	78/232 (33.62%)	154/232 (66.38%)	232/458 (50.66%)	Reference	Reference	Reference	Reference
Yes	24/226 (10.62%)	202/226 (89.38%)	226/458(49.34%)	4.26 (2.58–7.05)	<0.001^***^	3.09 (1.64–5.80)	<0.001^***^
Pleural indentation							
No	60/185 (32.43%)	125/185 (67.57%)	185/458 (40.39%)	Reference	Reference	Reference	Reference
Yes	42/273 (15.38%)	231/273 (84.62%)	273/458 (59.61%)	2.64 (1.68–4.14)	<0.001^***^	1.10 (0.60–2.03)	<0.001^***^
Nodule type							
Solid	86/274 (31.39%)	188/274 (68.61%)	274/458 (59.83%)	Reference	Reference	Reference	Reference
GGNs	3/45 (6.67%)	42/45 (93.33%)	45/458 (9.83%)	6.40 (1.93–21.34)	0.002^**^	28.25 (6.85–116.40)	<0.001^***^
Part‐solid	13/139 (9.35%)	126/139 (90.65%)	139/458(30.35%)	4.43 (2.37–8.29)	<0.001^***^	6.57(3.10–13.94)	<0.001^***^

*Note*: (1) The people with smoking included former and current smokers; (2) Group 45–60 covers 60 years; (3) Group 0.6–1.0 covers the maximum diameter is 0.6 and 1.0 cm; Group 1.0–2.0 covers the maximum diameter is 2.0 cm; Group 2.0–3.0 covers the maximum diameter is 3.0 cm.

^*^ *p* < 0.05.

^**^ *p* < 0.01.

^***^ *p* < 0.001.

### Nodule characteristics

The characteristics of the nodules according to lung cancer status are shown in Table 1. In a univariate analysis, significant consistent predictors of lung cancer not only included the age, but also covered the nodule size, lobulation, spiculation, satellite nodule, vessel convergence sign, pleural indentation and nodule type (*p* < 0.05).

We took the maximum diameter less than 0.6 cm as a reference, and found that an increase in nodule size was associated with lung cancer to some extent (0.6–1.0 cm, OR: 3.60; 95% CI: 0.78–16.63; *p* > 0.05; 1.0–2.0 cm, OR: 5.21; 95% CI: 1.21–22.49; *p* < 0.05; 2.0–3.0 cm, OR: 10.38; 95% CI: 2.32–46.54; *p* < 0.01). The location of a nodule was evaluated in terms of lobar distribution. A greater number of nodules and a higher number of cancers were observed in the right upper lobe than others. For this reason, the right upper lobe was compared with the other lobes in binary logistic regression analysis. Finally, we found that the right middle lobe (OR: 0.90; 95% CI: 0.37–2.20), right lower lobe (OR: 0.96; 95% CI: 0.49–1.89), left upper lobe (OR: 0.79; 95% CI; 0.42–1.47) and left lower lobe (OR: 0.63; 95% CI: 0.34–1.18) were protective factors for lung cancer, but this was not statistically significant (*p* > 0.05). In addition, whether the boundary was distinct was not statistically significant (*p* > 0.05) for judging whether the nodules were benign or malignant.

In our study, those nodules with lobulation (OR: 6.76; 95% CI: 3.54–12.91), spiculation (OR: 3.89; 95% CI: 2.34–6.47), vessel convergence signs (OR: 4.26; 95% CI: 2.58–7.05) and pleural indentation (OR: 2.64; 95% CI: 1.68–4.14) were at high risk of malignancy (*p* < 0.001). However, nodules with satellite nodules had a lower risk for lung cancer (OR: 0.06; 95% CI: 0.01–0.47; *p* = 0.008). It is worth mentioning that these nodules are rarely accompanied by satellite nodules (6/458), especially those found to be malignant (1/458). Previous studies have concluded that rheumatoid pulmonary nodules are more likely to have satellite nodules.^20^ We think it may better explain this phenomenon.

Among the 458 pulmonary nodules patients who had analyzable CT imaging, the majority of nodules were solid (59.83%) in appearance. GGNs and part‐solid nodules accounted for 9.83% and 30.35% of nodules, respectively. In the univariate analysis, GGNs (OR: 6.40; 95% CI: 1.93–21.34; *p =* 0.002) and part‐solid nodules (OR: 4.43; 95% CI: 2.37–8.29; *p* < 0.001) were risk factors for lung cancer.

### Predictors of malignancy

We removed the variables that were not significant in the univariate model, obtaining the multivariate model shown in Table 1, which includes the largest nodule diameter, lobulation, spiculation, satellite nodule, vessel convergence sign, pleural indentation, GGNs and part‐solid as all significant predictors of a nodule being malignant. Logistic regression analysis is essential in displaying how multiple variables act on each other and quantifying the effect size of each characteristic; however, it is unrealistic to put into use during the clinical diagnosis.

Machine learning models were constructed to distinguish lung cancer from benign lung diseases. First, all clinical characteristics were used as input features to develop the models of naivebayes‐1, SMO‐1, J48‐1 and randomforest‐1. Then, all clinical and imaging characteristics were employed as the input variables to develop the models of naivebayes‐2, SMO‐2, J48‐2 and randomforest‐2. Finally, clinical and imaging features were extracted from the logistics regression screening model and adopted to develop the models of naivebayes‐3, SMO‐3, J48‐3 and randomforest‐3. The effect of the model was evaluated by sensitivity, specificity, precision, F‐measure and AUC. For each model, we selected 3–10‐fold cross‐validation and in Table 2 we showed the average, maximum and minimum values. For example, three‐fold cross‐validation is that the dataset was randomized and split up into three subsets with similar class balances, then we used two subsets to train a model in each fold, while the remaining subsets were used to validate it. The SMO‐1 model determined all nodules as malignant. The evaluation of various models is shown in Table 2. Results showed that the efficiency of the naivebayes‐2 was higher than other models (*p* < 0.001) except randomforest‐2 (*p =* 0.996), naivebayes‐3 (*p =* 0.999) and randomforest‐3 (*p* = 0.002) by AUC comparison. However, the sensitivity (83.4% vs. 83.2%) and specificity (46.2% vs. 37.9%) of J48‐2 was slightly higher than naivebayes‐2. Attracted by the intuitiveness and visibility of the decision tree, although it is not the best classifier, we carried out a specific analysis of the decision tree. In terms of decision tree, each model achieved the largest AUC in the six‐fold verification. J48‐2 was better than J48‐1 (*p* < 0.001), and there was no difference between J48‐2 and J48‐3 by AUC comparison (*p* > 0.05). The J48‐2 model was too complicated to be applicable to the clinic, so in the end we chose J48‐3, hoping to give clinical doctors a reference.

**TABLE 2:** Effect evaluation of machine learning models

Models	Sensitivity	Specificity	Precision	F‐measure	AUC
NaiveBayes‐1	0.775 (0.762–0.786)	0.688 (0.667–0.695)	0.725 (0.697–0.750)	0.720 (0.707–0.730)	0.646 (0.624–0.663)
SMO‐1	‐	‐	‐	‐	0.500
J48‐1	0.754 (0.707–0.771)	0.745 (0.692–0.766)	0.661 (0.624–0.700)	0.685 (0.675–0.708)	0.561 (0.545–0.587)
RandomForest‐1	0.702 (0.681–0.716)	0.682 (0.655–0.728)	0.662 (0.634–0.677)	0.679 (0.655–0.693)	0.554 (0.539–0.563)
NaiveBayes‐2	0.832 (0.825–0.841)	0.379 (0.353–0.398)	0.823 (0.817–0.834)	0.826 (0.820–0.836)	0.821 (0.815–0.827)
SMO‐2	0.847 (0.832–0.858)	0.397 (0.363–0.440)	0.837 (0.819–0.850)	0.836 (0.818–0.849)	0.725 (0.696–0.746)
J48‐2	0.846 (0.825–0.869)	0.372 (0.336–0.413)	0.837 (0.816–0.863)	0.838 (0.818–0.861)	0.714 (0.668–0.747)
RandomForest‐2	0.844 (0.834–0.852)	0.444 (0.420–0.467)	0.835 (0.822–0.846)	0.828 (0.817–0.834)	0.816 (0.810–0.827)
NaiveBayes‐3	0.831 (0.825–0.836)	0.382 (0.362–0.399)	0.822 (0.817–0.827)	0.825 (0.820–0.830)	0.818 (0.814–0.820)
SMO‐3	0.835 (0.817–0.843)	0.496 (0.472–0.549)	0.828 (0.801–0.839)	0.812 (0.787–0.821)	0.670 (0.634–0.684)
J48‐3	0.834 (0.810–0.845)	0.462 (0.409–0.497)	0.822 (0.793–0.836)	0.817 (0.796–0.830)	0.741 (0.706–0.763)
Random Forest‐3	0.822 (0.808–0.834)	0.419 (0.390–0.451)	0.810 (0.799–0.824)	0.813 (0.803–0.827)	0.768 (0.758–0.790)

**FIGURE 2:**
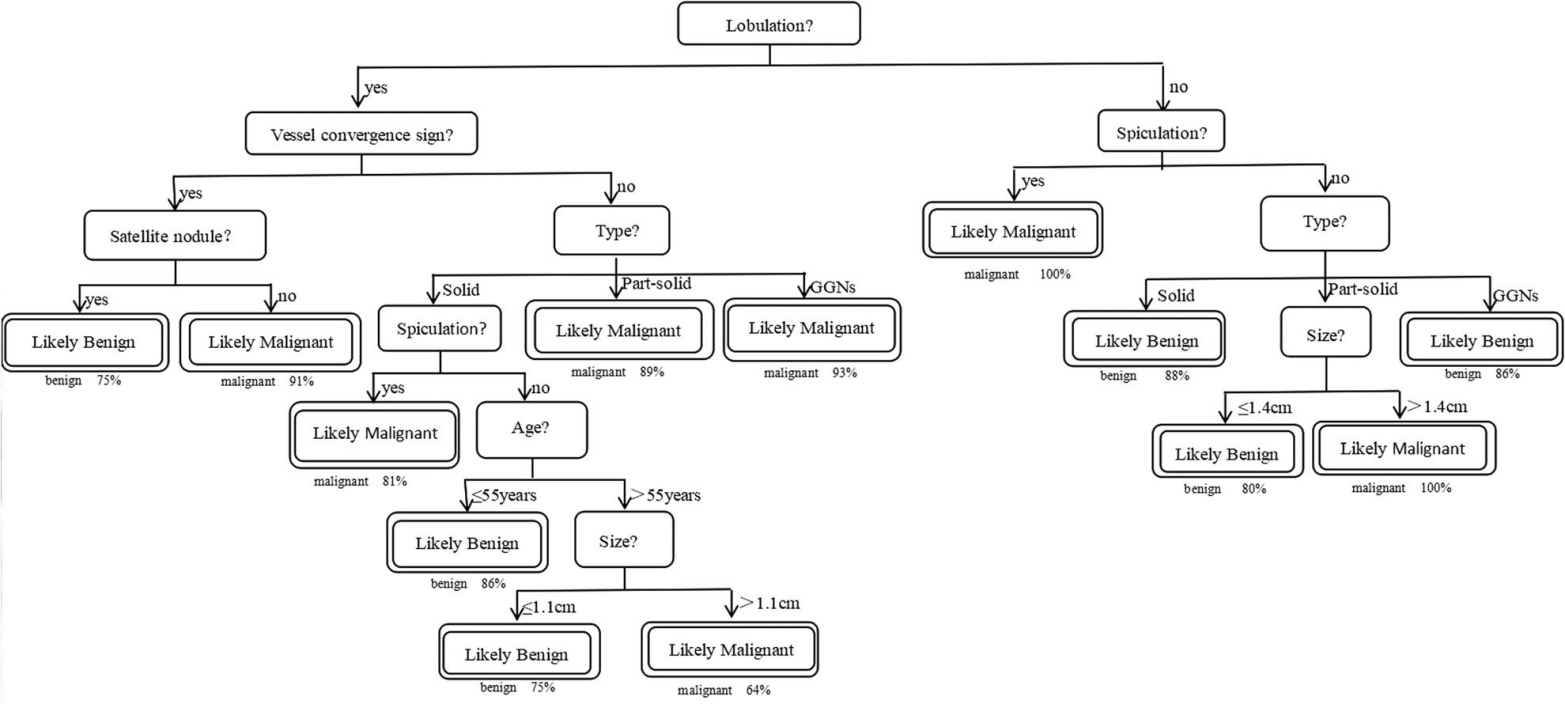
Proposed decision tree for prevalent nodules in a patient with pulmonary nodules

A streamlined version of this evidence‐based decision tree is shown in Figure 2. Per nodule, this classifier is 84.5% accurate overall. The decision tree shows that lobulation was assigned by the first and most informative node, followed by spiculation, vessel convergence sign, nodule type, satellite nodule, nodule size or patient age. The decision tree can be converted into a set of if‐then rules by tracing the path from the root node to each terminal node. The if‐then rules created by the model are presented in Table 3.

**TABLE 3:** Thirteen if‐then rules extracted from the decision tree in Figure 2

Rule 1: IF without lobulation, without spiculation, solid, THEN nodule is benign (22/25 or 80%)
Rule 2: IF without lobulation, without spiculation, part‐solid, size ≤ 1.4 cm, THEN nodule is benign (4/5 or 80%)
Rule 3: IF without lobulation, without spiculation, part‐solid, size > 1.4 cm, THEN nodule is malignant (4 or 100%)
Rule 4: IF without lobulation, without spiculation, GGNs, THEN nodule is benign (6/7 or 86%)
Rule 5: IF without lobulation, with spiculation, THEN nodule is malignant (4 or 100%)
Rule 6: IF with lobulation, without vessel convergence sign, solid, without spiculation, age ≤ 55, THEN nodule is benign (19/22 or 86%)
Rule 7: IF with lobulation, without vessel convergence sign, solid, without spiculation, age >55, size ≤ 1.1, THEN nodule is benign (3/4 or 75%)
Rule 8: IF with lobulation, without vessel convergence sign, solid, without spiculation, age >55, size > 1.1, THEN nodule is malignant (25/39 or 64%)
Rule 9: IF with lobulation, without vessel convergence sign, solid, with spiculation, THEN nodule is malignant (43/53 or 81%)
Rule 10: IF with lobulation, without vessel convergence sign, part‐solid, THEN nodule is malignant (41/46 or 89%)
Rule 11: IF with lobulation, without vessel convergence sign, GGNs, THEN nodule is malignant (27/29 or 93%)
Rule 12: IF with lobulation, with vessel convergence sign, without satellite nodule, THEN nodule is malignant (197/216 or 91%)
Rule 13: IF with lobulation, with vessel convergence sign, with satellite nodule, THEN nodule is benign (3/4 or 75%)

## DISCUSSION

Our study analyzed the differences in clinical and CT imaging characteristics between benign and malignant nodules, determined the increased odds ratio (OR) of lung cancer among patients with pulmonary nodules and provided a more feasible method for judging the nature of nodules in clinical work.

Moreover, a previous study^21^ has shown that the combined use of multiple methods to build a model can optimize the model. To our knowledge, this is the first study that has examined and combined the utility of logistic regression model with the machine learning models, the naivebayes, decision tree, support vector machine and random forest model, to predict lung cancer in a large Chinese population. Through our comparison, the decision tree model has certain advantages. We have tried to establish a variety of decision tree models to screen out the optimal model.

Decision tree is a valuable classification algorithm in data mining methods.^22^, ^23^ In the decision tree, the first variable (root) is the most important factor and variables far away from the root are the next important factors in classifying the data.^24^ This study shows that lobulation is the most significant attribute discriminating between benign and malignant nodules. A lobulated border was defined when a portion of the surface of a lesion showed a wavy or scalloped configuration, apart from regions abutting the pleura.^25^ This result once again confirms that the feature of lobulation in previous studies^25^, ^26^, ^27^ is a predictor of malignant nodules.

The size and morphology of a pulmonary nodule are the two primary determinants of cancer risk.^7^ Morphology refers specifically to the margins (smooth, lobulated, or spiculated) and attenuation (solid, partly solid, or purely ground‐glass) of the nodule.^28^ A fine spiculated margin is defined as very fine linear strands extending radially 1–2 mm beyond a lesion.^25^ The decision tree shows that spiculation, vessel convergence sign, nodule type, satellite nodule, nodule size and age of patient are the following important factors after lobulation. The tree identified a subgroup of individuals (22 nodules [88%]) without lobulation, without spiculation and solid that were benign nodules. Another subgroup of individuals (197 nodules [91%]) with lobulation, with vessel convergence sign and without satellite nodule were identified as malignant nodules.

Notably, in the decision tree, we found that the sizes of 1.1 and 1.4 cm were also the dividing points, which may differ slightly across different samples and patient populations, and this tree provides an outline on how to estimate malignancy risk. However, the conclusion coincides with previous studies,^27^, ^28^ that nodules of greater diameter are more likely to be malignant. A previous studies indicates that the lifetime risk of receiving a diagnosis of cancer by age 30 years is approximately 1% and is 2% by age 40 years.^29^ We also found that the average age of a lung cancer diagnosis is 58.91 ± 9.69 years old, and the risk of suffering from lung cancer increases with age, which is consistent with many previous studies.^30^, ^31^ The decision tree confirms that 55 years old is a truncation, and we therefore advocate the use of routine chest CT scans for older individuals.

A major strength of this study is that we used a real medical dataset of patients with lung nodules who underwent VATS lobectomy at Shandong Provincial Hospital. All laboratory pathological results were obtained in all patients, and the results are therefore more reliable. Through the AUC evaluation, the naivebayes model has obvious advantages, but the simplicity and visualization of the decision tree make it possible for use by clinicians. The selection of features in logistic regression make the use of decision trees easier. In future, our model does need prospective trials to be validated in a larger patient population.

Our study has limitations. First, the sample selected were patients undergoing VATS lobectomy, and our study is not applicable to patients with advanced metastatic lung cancer. Second, data were collected from only one large hospital in China. Further studies with additional data from this hospital and other centers need to be performed. Third, the clinical trials of patients were based on medical records; therefore, it may lead to an information bias.

In conclusion, in comparison to previous studies, our study had a much larger sample size of nodules (458 patients) and each sample was from PACS and based on pathological results, which allowed us to generate a more accurate and robust model. Here, we combine decision tree with logistics regression, simplifying the model as much as possible without reducing the goodness of fit of model, and thus making it possible to use clinically, especially for young doctors who do not have extensive experience in judgment. Although our decision tree is not specific enough, it provides a new concept for our future clinical work and research, hopefully enabling better use of CT in the early screening of lung cancer.

## CONFLICT OF INTEREST

The authors declare that they have no competing interests.
